# Physiological Candles

**Published:** 1889-10-26

**Authors:** 


### The Doctor's Pulpit.

### PHYSIOLOGICAL CANDLES.

### Development.

There is a time in the history of every human being when his whole substance consists of a little mass of protoplasm, hardly to be distinguished by the naked eye from a slice of the pulpy part of an oyster. There is a time when the child has a brain but cannot think, a musical faculty but cannot sing, a soul but cannot worship ; when he has legs that cannot walk, arms that cannot carry burdens, a tongue that cannot taste, and a nose that cannot smell. Yet in a few short years the child becomes the wild and ubiquitous schoolboy, who can run swiftly as the wind, wrestle with his companions in tremendous tugs of war, work out problems in mathematics, and sing with infinitely more sweetness, because with infinitely more soul, than the rarest nightingale that Kentish orchards ever gave home to. What may the same child do when he is a grown man ? The scientific work of a Newton, the moral uplifting of a Carlyle, the fighting of a Wellington, or the statesman's duty of a Burleigh or a Pitt.

That may possibly sound somewhat inflated to the cynics of a cynical age. But can any man, cynic or otherwise, say that it is more than the sober truth ; the protoplasm does become the child, the child the man, and the man the genius—to-day—exactly as in earlier and more heroic times. "But we are all for the daylight of reason, and the hard facts of science, and not at all for rhetoric or poetry," say some. We, too, are for the pure light of reason, though we see no just cause why it should be particularly "dry" ; and for the genuine facts of science, although we quite deny that they are wholly or mainly "hard," when they are properly understood. This "development" of the protoplasm into the child, and of the child into the man, is altogether a lively, moving, amusing, working, playing, and wonderful business, and has as little of the "dry" light and the "hard" fact about it as can well be imagined.

But in what sense may the fact of development be called a "candle," either physiological or otherwise ? In the sense that it is one of the most brilliant illuminators of dark places that the world has ever seen or known. The idea of development is a kind of beneficent Pandora's box, out of which all kinds of helpful and guiding ideas may be constantly made to fly. A man has but to ask himself, "Is there in human affairs such a principle as is described by the doctrine of development, and have I any kind of control of that principle ?" He has but to answer himself in the affirmative, and to believe firmly in his own affirmation, in order to become master at once of one of the greatest sources of power known in the world. In human affairs, as well as in those of the world at large, a movement once commenced tends to grow and increase : a force set in motion gathers energy in its progress. That is a fact of the very primest significance both on the positive and the negative sides. Does a severe catarrh, for example, begin in a man's nose ? It ordinarily extends, in the most natural way, from the nose to the throat, from the throat to the windpipe, and from the windpipe to the bronchial tubes. Here is a man with shooting pains in his head, which, besides being hard to bear, depress his spirits and make him unfit for work. His hands tremble and his lips look irresolute. He is ten years older than his age. Twenty years ago he was one of the strongest of the strong ; could stand a stiff day's hunting or shooting, a hard night's drinking, or a prolonged and exhausting period of mental labour with the best. Now, at fifty, he can't stand neither shooting nor drinking, and so far from being able to perform any exhausting mental labour, he cannot even write a letter until he has had a strong glass of brandy and water to steady his hand and give a little artificial tone to his mind. He says he never felt any of these distressing symptoms until two or three years ago. No ; but he started off his organs on a course of development by his unwholesome habits twenty-five years ago, and the result is what we see to-day. Scientific persons, who love to be painfully precise, would say that this is not "development," but "retrograde metamorphosis." In one sense, however, inasmuch as it is the production of new and non-natural effects by means of definite and positive causes, it is as truly a "development" as it is a "retrogade metamorphosis." The motion was commenced twenty-five years ago in a way so small as to be absolutely imperceptible to the finest instruments of the physiologist, or even to the sensibilities of the man himself. To-day the passer-by in the street can see that drink has effected a ruin in that iron constitution which no skill of man can ever again repair.

There would be no difficulty whatever in illustrating from the innumerable life-histories of mankind the positive aspect of development—what scientists would call development proper. A feeble, puny, and diseased child, by means of food of the highest nutritive value, of abundance of pure air and active tissue metamorphosis, of judicious exercise and sound sleep, has become a man or a woman of energetic heart, active liver, sound digestion, and a clear and capable brain. He was started off in the right direction ; and Nature, grateful for the trust reposed in her, set the principle of development at work ; and it used up all the resources supplied by the wisdom of the parent or physician : with the result that the puny child of thirty or forty years ago is the muscular, intellectual, and moral giant of to-day.

There is in nature, what Mr. Matthew Arnold has called in another field of thought, a "stream of tendency," that makes for development and growth. That everybody knows and admits. What people almost universally fail to appreciate is that in their own personal affairs this stream of tendency may be absolutely depended upon as a making or marring force. It is always in movement and activity ; and every single act of a man's life is used up by it, either in the making or marring of himself. It acts in the region of the body and of every organ of the body ; it acts in the region of the mind and of every faculty and power of the mind. Few people ever dream of what they might become, either in health or in disease, and that with little or no effort on their own part beyond a steady set of the mind and purpose in an upward or downward direction. Similarly, not one in ten thousand has the forethought to perceive that a resolute set of the mind in the direction of all that he can understand of what is highest and noblest will inevitably lead him, in course of time, to a steady and rocklike nobility of character which will be the wonder of his contemporaries and still more of himself. On the other hand, the least deflection of mind and morals downwards will as inevitably bring a man to a condition which, in his earlier days, he would not have considered possible. It is all natural—quite natural. It is a scientific law of man. The truth is we do not take ourselves half seriously enough. We do not trouble to understand ourselves scientifically. The present age is full of the doctrine of development; but only as it relates to the lower animals, or to man in his prehistoric or savage state. But the development that is going on to-day is the true "time-spirit" that most concerns the men and women of to-day. The development of past ages is merely historically interesting and curious. The development of to-day is doing or undoing the work of the statesman, of the philosopher, of the religious teacher, of the physician, of everyone who can think for or desire his own or any other man's well-being. Development, like electricity, though not understood, can be laid hold of and utilised by the physician, by the statesman, by the moralist, by every man for himself. Talk and speculation are the bane of all thinkers, scientists, as well as others. It is when thought weds fact, when the broadest, deepest, and highest convictions of men are realised to their fullest possibility in doing duty, that development takes its noblest form of progress, expansion, strength, and worth. Knowledge to-day is every man's possession by the bushel and the cartload. High and noble conduct is as rare as it was a thousand years ago; and so development is lateral or downwards almost universally, and only upwards fitfully and in solitary instances—instances that are so uncommon as to be almost generally disbelieved.

